# Hand-Book of Inorganic Chemistry, for the Use of Students

**Published:** 1856-12

**Authors:** 


ART. V.—*Hand-Book of Inorganic Chemistry, for the Use of Students.* By WM. GREGORY, M. D., F. R. S. E., Prof. of Chemistry in the University of Edinburgh, and author of "Hand Book of Organic Chemistry." Fourth American, from the third English edition. To which is added The Physics of Chemistry. By J. MILTON SANDERS, M. D., LL. D., Professor of Chemistry in the Eclectic Medical Institute, of Cincinnati; Member of the American Association for the Advancement of Science. New York: A. S. BARNES & Co., 51 and 53 John Street. 1857.

Can anything good come out of Nazareth? The Eclectic school has thrown itself into the book-market with great energy. As a general thing, their books are an odd mixture of science and humbug. This expression does not apply to the work before us. Dr. Gregory's Chemistry was a very excellent work, but was deficient in the physics of the science. This deficiency has been supplied by Dr. Sanders, and though we have a proclivity to find fault with anything from that source, it would be unjust, in this brief acknowledgment of the reception of the work, to fail to say that Dr. Sanders has added materially to the value of this edition. His paper on photography, the only one which we have read, is excellent in style of description and in practical character.

